# Laser light routing in an elongated micromachined vapor cell with diffraction gratings for atomic clock applications

**DOI:** 10.1038/srep14001

**Published:** 2015-09-14

**Authors:** Ravinder Chutani, Vincent Maurice, Nicolas Passilly, Christophe Gorecki, Rodolphe Boudot, Moustafa Abdel Hafiz, Philippe Abbé, Serge Galliou, Jean-Yves Rauch, Emeric de Clercq

**Affiliations:** 1 Micro-Nano Sciences & Systems department, FEMTO-ST Institute, UMR 6174 CNRS and Université de Franche-Comté, Besançon, France; 2 Time & Frequency department, FEMTO-ST Institute, UMR 6174 CNRS and Université de Franche-Comté, Besançon, France; 3 LNE-SYRTE, Observatoire de Paris, CNRS, UPMC, Paris, France

## Abstract

This paper reports on an original architecture of microfabricated alkali vapor cell designed for miniature atomic clocks. The cell combines diffraction gratings with anisotropically etched single-crystalline silicon sidewalls to route a normally-incident beam in a cavity oriented along the substrate plane. Gratings have been specifically designed to diffract circularly polarized light in the first order, the latter having an angle of diffraction matching the (111) sidewalls orientation. Then, the length of the cavity where light interacts with alkali atoms can be extended. We demonstrate that a longer cell allows to reduce the beam diameter, while preserving the clock performances. As the cavity depth and the beam diameter are reduced, collimation can be performed in a tighter space. This solution relaxes the constraints on the device packaging and is suitable for wafer-level assembly. Several cells have been fabricated and characterized in a clock setup using coherent population trapping spectroscopy. The measured signals exhibit null power linewidths down to 2.23 kHz and high transmission contrasts up to 17%. A high contrast-to-linewidth ratio is found at a linewidth of 4.17 kHz and a contrast of 5.2% in a 7-mm-long cell despite a beam diameter reduced to 600 μm.

For more than fifty years, atomic clocks, relying on a well-defined atomic transition to discipline the frequency of a local oscillator, have provided the most stable and accurate time and frequency references^1^,^2^,^3^. During the last decade, miniature atomic clocks, based on microfabricated alkali vapor cells, have been developed to provide alternative frequency references^4^,^5^, more compliant with mobile applications and exceeding the stability reached by the most evolved quartz oscillators. Many applications such as navigation or telecommunications could greatly benefit from battery operable atomic references. However, the stringent requirements imposed by such applications require drastic reduction in their size and cost while maintaining high performances.

The miniaturization of atomic clocks has been accomplished thanks to several breakthroughs. First, the discovery of the coherent population trapping (CPT) phenomenon^6^,^7^ has revealed the ability to interrogate the alkali vapor with an optically carried microwave signal, which sidesteps the need for bulky microwave cavities. Then, power-efficient vertical cavity surface-emitting lasers (VCSELs) providing high modulation capabilities have replaced consuming discharge lamps^8^. Finally, micromachining technologies have provided solutions for the batch fabrication of much smaller vapor cells at low cost^9^. Those advances opened the path to the first chip scale atomic clock (CSAC) demonstration^4^,^5^, and the first commercialized product shortly later^10^.

In its most basic configuration, a CSAC consists of a straight-lined arrangement in which a VCSEL beam is circularly polarized by a quarter-wave plate (QWP) and illuminates one side of an alkali vapor cell^11^. The transmitted light is then collected by a photodiode on the other side of the cell. The VCSEL is modulated to produce two phase-coherent optical lines. CPT occurs when the frequency difference of those lines matches the ground-state hyperfine splitting of the atoms. At resonance, atoms are pumped into a coherent dark state in which they theoretically no longer interact with the light fields. As a result, a peak of transparency appearing at the bottom of a homogeneously broadened optical line can be detected by the photodiode. A static magnetic field is established in the cell to lift the Zeeman degeneracy and allow to address the magnetic field insensitive clock transition.

The short-term fractional frequency stability of a clock is improved by narrowing the CPT signal and increasing its height. The signal is generally narrowed by adding a buffer gas in the cell to confine alkali atoms and extend their interaction time with the light^12^.

In order to preserve the CPT signal quality and hence the resulting frequency stability, three main aspects regarding the illumination schema should be addressed. Those aspects include a sufficient collinearity between the propagation direction of the probing light beam and the static magnetic field axis, a highly circular polarization state and a large and uniform illumination of the volume occupied by the alkali vapor so that most of the atoms are likely to contribute to the CPT signal^13^,^14^. Nevertheless, fulfilling all these constraints can be difficult when it comes to miniaturizing the device down to millimetric scales.

In addition to being compact and comply with those illumination requirements, the device must remain easy to assemble. As for many MEMS based products, the assembly can indeed be predominant in the overall cost. The integration of components at the wafer level rather than at the package level becomes necessary and can tip the balance in the compromise that is usually conceded between stability, compactness and integrability. Aiming at improving some of these aspects, alternative configurations using additional micro-optical elements such as reflectors or microlenses have been proposed. However, few of them actually address all those constraints.

This paper reports on an original architecture where laser light is routed in an alkali vapor cell with angled reflectors formed in a wet-etched silicon cavity and integrated diffraction gratings. This architecture, in accordance with the illumination requirements to improve the CPT signal quality, provides both compact beam shaping ability and a simpler integration of the laser and the detector which can be placed side by side on the same electronic plate. These features allowing an extensive wafer-level approach could answer the need for cost and size reduction. In addition, we show that an elongated geometry does not jeopardize the performances, quite the contrary, since the results exhibit a potential for stability improvement. This novel approach based on an elongated cell is not straightforward. Indeed, it does not allow the reduction of the CPT resonance linewidth because of the dominating sidewalls collision rate. But the contrast is significantly improved, balancing the use of both a tinier cavity and a smaller beam, and improving the figure of merit.

The first section reviews different existing optical designs used in CSACs and describes specifically the design and fabrication of the grating based cell. The second section describes a basic model and the CPT spectroscopy results are discussed in the light of the model predictions.

## Materials and Methods

### Architectures

**Figure 1:**
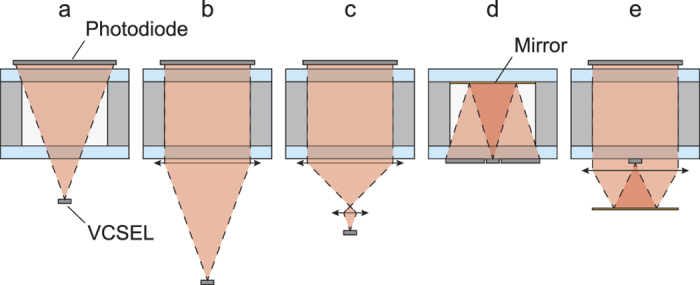
Sketches of optical configurations (not to scale). (**a**) Naturally diverging beam. (**b**) Simple collimation. (**c**) Collimation and reduction of the propagation length with a lens doublet. (**d**) Folded and diverging beam. (**e**) Collimation and reduction of the propagation length by beam folding using a mirror and a dual-focus lens.

The most basic optical assembly of a CSAC essentially consists of a VCSEL, an alkali vapor cell and a photodiode arranged so that the optical train is straight-lined (Fig. 1a). The natural divergence of the VCSEL allows to reach a sufficient beam diameter in the cell provided that the distance between them is long enough. This simple approach is particularly appealing since no optical element is added (except for a QWP and a neutral density, not shown in Fig. 1 because they do not have a beam-shaping function). This architecture was adopted by Lutwak *et al.* in the first commercial device^10^. However, depending on the cell diameter, several millimeters can be required to reach the desired beam width while reducing the light intensity inhomogeneity and ensuring collinearity between the light propagation and the magnetic field.

Another approach involves a collimating lens located close to the entrance window of the cell (Fig. 1b). Even though it provides a beam collinear with the magnetic field, a large and uniform illumination still requires a long propagation distance^4^. In order to shorten the beam shaping distance and thereby the assembly length, a lens can be added before the collimating lens to increase the divergence and form a beam expander (Fig. 1c). While this solution can collimate a large beam in a relatively tight space, it comes at the expense of constraining alignments during the assembly of the first high numerical aperture lens of the doublet^15^,^16^.

Configurations based on reflectors have also been proposed to reduce further the assembly size by ‘folding’ the optical path. For instance, Lutwak *et al.* also proposed a folded configuration in which the beam performs a round trip through the alkali vapor cell, with the addition of a mirror on the former exit window (Fig. 1d)^17^. In this case, the VCSEL can be placed at the center of a surrounding photodetector on the same electronic plate, offering a significant advantage regarding the assembly of the physics package. However, this specific configuration led to an ‘inhomogeneous light shift’ due to a highly uneven illumination within the cell, both in direction and intensity. Moreover, it reduces significantly the density of VCSELs that can be fabricated on a wafer and thus the cost efficiency. The design was finally changed to a non-folded and non-collimated design (Fig. 1a) which provides better performances and reduces the cost of the optoelectronic components^18^.

Slightly later, DeNatale *et al.* proposed a design in which the VCSEL is oriented outward the cell. The beam is reflected back toward the cell with an additional mirror (Fig. 1e)^19^,^20^. In this design, a dual-focus optic allows to increase the divergence of the beam just emitted from the source and collimates the beam reflected by the mirror at the same time. This solution is similar to the configuration using a doublet (Fig. 1c) but in a folded arrangement where the two lenses are part of a single element. This component joining a Fresnel lens with a microlens at its center can be batch fabricated and is more compliant with wafer-level alignments.

The common feature of the designs presented so far is a vapor cell fabricated by sandwiching a silicon wafer featuring a dry-etched through-cavity between two glass wafers. The resulting cell length is limited by the deep reactive ion etching (DRIE) which can hardly reach 2 mm depths^21^,^22^. In order to overcome this limitation, the interrogation beam can instead be propagated in a cavity along the wafer surface. Even if the cell diameter is now limited by the wafer thickness, its length can be extended at will. The number of probed atoms can thus be increased while keeping a small beam diameter, which relaxes the beam shaping requirements. From the integration standpoint, it is desirable to keep the VCSEL emission direction and the detector orientation normal to the wafer plan, hence, the light beam should be folded accordingly.

Reflectors can be patterned on the angled sidewalls of a silicon cavity. The most appealing approach is to take advantage from the surface quality of (111) crystallographic planes revealed by the anisotropic wet etching of (100)-oriented silicon wafers. Unfortunately, because of the diamond structure of silicon, these crystallographic planes are oriented at 54.74° from the wafer surface which differs from the ideal 45° orientation. The angle of the input beam should therefore be corrected before the reflection. Based on such reflectors, Youngner *et al.* proposed a solution where the interrogation beam propagates along the wafer surface across a suspended cell^23^,^24^. This design uses prisms structured in glass to refract the beam and correct the angle of incidence on the reflector so that its reflection is in the wafer plane. However, the batch fabrication of such a refractive component is challenging. In addition, the vapor is contained in a smaller portion than the volume available between the two reflectors and the fact that the QWP is placed between the reflector and the vapor cell makes it difficult to integrate at the wafer level. Another solution based on refractive components could also rely on a misaligned microlens but its behavior would be very sensitive to alignments during packaging and the circular polarization state could be quite affected.

**Figure 2:**
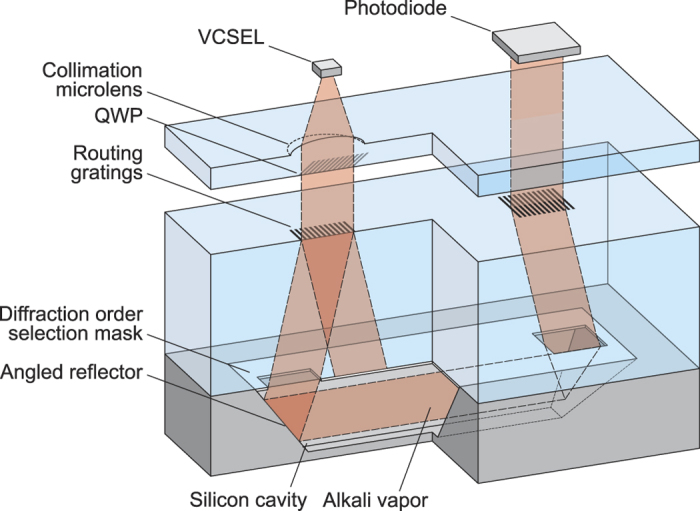
Principle of an alkali vapor cell based on diffraction gratings.

In this work, we consider the integration of diffraction gratings on an alkali vapor cell in order to route the probing laser beam and perform the required angle correction. Unlike a microlens, the advantage is that gratings will perform the same optical function regardless of the incident beam lateral position. Concerning the cell, the whole cavity between the reflectors contains alkali vapor and its length can thus be freely extended. The circular polarization is preserved by the gratings which enable to integrate a QWP upstream at the wafer level since the incident beam remains under normal incidence. This solution provides both compactness and a simpler integration of the laser and the detector which can be placed side by side on top of the cell (Fig. 2).

### Gratings design and fabrication

**Figure 3:**
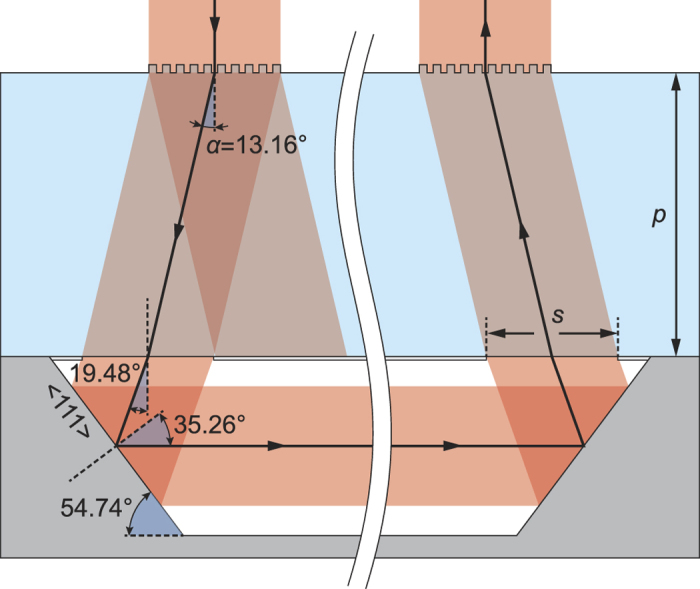
Routing of the beam in the cell.

The optical function of the diffraction gratings is to diffract most of the energy in the first diffraction orders. Their diffraction angle is fixed by the 54.74° orientation of the silicon planes used as reflectors. To ensure a beam propagation along the wafer, the incident angle on the reflectors should thus be 35.26°, which corresponds to an angle of 19.48° at the glass window exit (Fig. 3). The angle *α* of the beam within the glass window is deduced from Snell law. In our case, the cell is sealed with a borosilicate glass whose refractive index is *n* = 1.4645 at *λ* = 894.6 nm. The angle *α* is then equal to 13.16°. The angles between the normal of the gratings and the propagation direction of the different diffraction orders obeys the grating equation. For the first diffraction order to be diffracted at *α* = 13.16°, the period of the grating should be 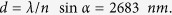 As electron beam lithography is used, the gratings period is controlled within a few nanometers.

Different types of gratings could perform this function. Blazed gratings appear as a suitable option as they diffract most of the energy into one diffraction order. However, for high diffraction angles, the efficiency drops down to ca. 65% due to shadowing effects^25^. Furthermore, the energy is dissipated in various orders, especially the second and third ones. In order to increase this efficiency at higher angles, binary blazed gratings with sub-wavelength features are good candidates^26^. Nevertheless, they are characterized by high aspect ratios and are consequently challenging to fabricate. Geometries slightly easier to fabricate could be considered^27^ but they also show sizeable difference in efficiencies depending on the incident polarization orientation and might not be appropriate for the conservation of a circular polarization state. Indeed, the grating should also conserve the beam circular polarization state, as required for the CPT spectroscopy. For this purpose, polarization diffraction gratings^28^ are very attractive, especially because they can break the efficiency of the scalar-domain limit and reach very high efficiencies^29^. In here, theoretical 100% diffraction efficiency of the first order where the incident circular polarization is conserved is achieved. Since the first demonstration based on liquid crystals^30^, polarization gratings made for infrared light^31^ and visible light were fabricated^32^. Conservation of circular polarization was also shown recently based on twisted nematic liquid crystals^33^. Unfortunately, despite this quasi-perfect behavior, polarization gratings require paraxial domain, which is no longer the case if the angle of diffraction is high. Indeed, enough sub-wavelength periods are required within each full-rotation period and, consequently, the latter cannot be as small as a few microns.

Lamellar gratings with binary corrugation were finally considered as they are easier to fabricate. First, the parameters maximizing the energy in the first order while minimizing it in the zeroth order had to be determined. Such type of gratings have been used e.g. as a phase mask to print periodic structures with characteristic dimensions of several tens of nanometers based on the interferences between the two ±1^st^ transmitted orders^34^. In addition to zeroth order cancellation, the incident circular polarization state should be preserved. All these conditions make it necessary to find the pair height (*h*) and fill factor (*f*) (corresponding respectively to the corrugation depth and the duty cycle) achieving the following characteristics: a cancelled zeroth order considering incident polarizations both parallel (transverse-electric TE) and perpendicular (transverse magnetic TM) to the grating vector, a maximized first order while keeping both TE- and TM-efficiencies equal and a minimized phase shift between the transmitted components in the first order. It can be noted that such a rather high period-to-wavelength ratio, imposed by the restricted angle of diffraction, implies that higher orders than the first one exist in the substrate (until ±4^th^) although their amplitudes are kept low.

**Figure 4:**
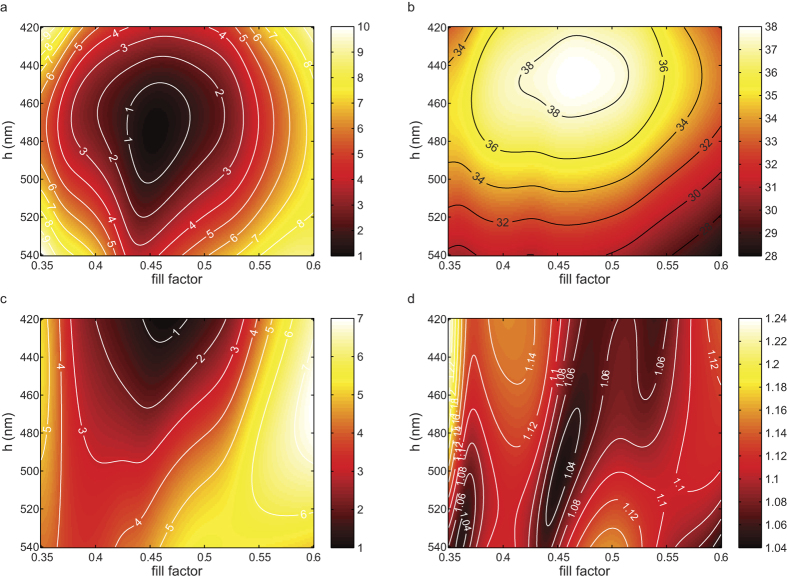
(**a**) Average of TE and TM efficiencies (%) in 0^th^ transmitted order, (**b**) average of TE and TM efficiencies (%) in 1^st^ transmitted order, (**c**) average of TE and TM efficiencies (%) in 2^nd^ transmitted order. (**d**) Ellipticity of the beam diffracted into the 1^st^ orders.

The gratings are made of Si_3_N_4_ stripes (*n* = 2.0064 at λ = 894.6 nm) on top of a borosilicate substrate in order to reduce the aspect ratio of the structure to be fabricated. The rigorous Fourier modal method is employed to compute the optimal parameters^35^. Figure 4a–c shows the efficiencies of the 3 first orders (0^th^, 1^st^ and 2^nd^, respectively). Note that we consider on the plot the average values between the two polarization components. In addition, the ellipticity of the beam diffracted into the first order is shown in Fig. 4d. The ellipticity is simply defined as the ratio between the maximum and the minimum of the intensity that would be recorded after a rotating polarizer located on the optical path of the 1^st^ order, i.e. as the ratio between the major and minor axis of an elliptical polarization. Therefore, a circular polarization corresponds to an ellipticity equal to unity.

As we can see from Fig. 4, there are no ideal parameters. Indeed, the best extinction of zeroth and second orders and the maximum of the 1^st^ order along with the minimum of ellipticity do not match perfectly. Fortunately, a suitable trade-off is found for *f* = 0.47 and *h* = 466 nm for which the ellipticity is only 1.04, and the transmission efficiencies are 0.54%, 38.05% and 2.40% for the 0^th^, and each of the 1^st^ and 2^nd^ orders, respectively. The slight ellipticity is mostly attributed to the difference between the transmission coefficients, although only equal to 0.7%, rather than to the phase shift that is close to zero. Tolerances in the range of ±20 nm on the height and ±0.02 on the fill factor values (i.e. ±53 nm on the linewidth) ensure an ellipticity better than 1.1 and a transmission over 36% in each of the first orders.

Hence, in order to discriminate the two efficient first orders, a mask, whose apertures match the grating size, is added on the bottom side of the lid. Depending on the grating lateral size *s*, a sufficient distance of propagation is required before the discrimination aperture (*p* ≥ *s*/(2 tan *α*)). In this work, we use commercially available borosilicate wafers whose thickness are fixed. The gratings size *s* was chosen to be 600 μm and *p* = 1.3 mm. Note that the alignment performed at wafer level between the cavity (reflectors), the aperture and the grating ensures that light normally incident to the grating will be routed inside the cavity. The orientation of the grating lines, perpendicular to the cavity axis, is also well controlled during fabrication.

A first set of gratings was actually designed and tested at 633 nm for optical characterization convenience. We employed electron beam lithography to fabricate 2 mm × 2 mm test structures. The latter behaved as expected and diffracted 37.7% and 38.6% in the two first orders oriented at 19.4° ± 0.3°. The ellipticity was measured to be 1.12 and 1.14, respectively. Note that the ellipticity of the incident beam was already 1.04. Meanwhile, the zeroth order was nearly suppressed (0.6%) and the second orders efficiencies were reduced to 5.5%. It has to be noted that the ellipticity in the zeroth and second orders was measured to be over 20 and 7 respectively, showing that the circular polarization conservation is only achieved in the 1^st^ order. Once the process optimized, the parameters were transposed to 894.6 nm which is resonant with the D_1_ line of cesium atoms.

### Cell fabrication

The first step of the cell fabrication consists in patterning the silicon cavity, while ensuring that the obtained sidewalls are mirror-like. Generating flat, large and optically smooth surfaces by etching is not straightforward and it has been the subject of previous studies^36^. A Cr/Au etch mask is patterned on a 100 mm silicon wafer with a 1.5 mm thickness and a (100) orientation. It is then immersed in a KOH solution at a concentration of 40% and heated at 70 °C for several hours to obtain a 650 μm deep cavity (0.5 μm min^−1^). This wet-etched cavity is referred to as the optical cavity. Next to the optical cavity, a second cavity is then etched using DRIE. This cavity aims at holding a solid compound of cesium called dispenser from which pure cesium vapor is released later on in the process. Channels connecting both cavities are patterned by DRIE along with the dispenser cavity.

As a bare silicon surface absorbs around 68% of the incident light at the considered wavelength, a coating should be deposited on the angled sidewalls to reach a high reflectivity and avoid deterioration of the polarization state due to dichroism (the ellipticity after one reflection under such incidence would be equal to 1.6). A solution based on silicon reflectors was also sought by Kitching *et al.* to achieve a crossed beam configuration for an atomic magnetometer^37^. A multilayer dielectric coating deposited on the silicon sidewalls to efficiently reflect circularly polarized beams was implemented by Perez *et al.*^38^. Indeed, dielectric mirrors are preferable over metallic mirrors since they do not affect the magnetic field and do not react with alkali metals. Although slightly less efficient, the reflectors of the cells presented here were coated with aluminum (the efficiency is reduced to 89% instead of 98% with dielectric multilayers and the ellipticity of the circular polarization is degraded to 1.2 after the first reflection).

After inserting a dispenser in the second cavity, the cell was sealed with a borosilicate glass lid using anodic bonding. So as to operate in the Dicke regime, a buffer gas (neon) was introduced in the chamber during the bonding following a procedure previously reported^39^,^40^. After the bonding, the dispenser was locally heated with a high power laser diode focused through the glass window, which releases cesium vapor in the cell.

**Figure 5:**
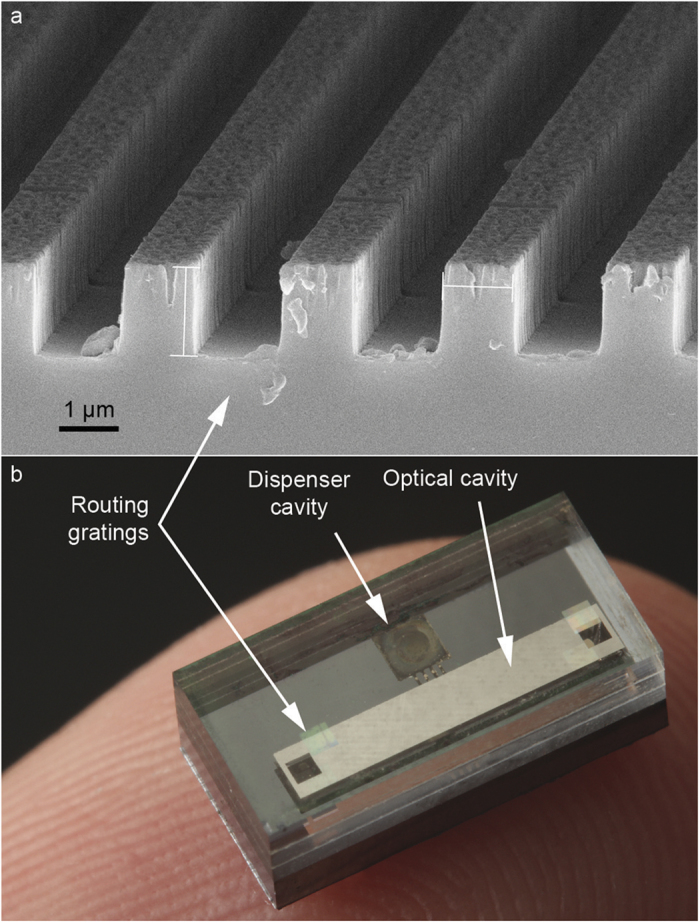
(**a**) SEM image of diffraction gratings. (**b**) Picture of a cell featuring diffraction gratings.

The gratings were patterned on a separate glass wafer. Two different processes were developed. The first approach consists in depositing a 470 nm thick Si_3_N_4_ layer using plasma-enhanced chemical vapor deposition (PECVD). A layer of resist is patterned using electron-beam lithography. The pattern is then etched with reactive ion etching (RIE) defining stripes in the silicon nitride layer. In the alternative approach, the gratings are directly etched in a glass wafer. A chromium etching mask is evaporated after the lithography. A lift-off is performed, removing the resist. The remaining metal stripes are then used as a mask to etch directly 1-μm-deep stripes in the glass wafer with RIE (Fig. 5a).

The metallic aperture which selects the first diffraction order was deposited on top of the lid after the anodic bonding, although it could be integrated on the inner surface of the cell if accurate alignment can be performed during anodic bonding.

The grating wafer is finally aligned and bonded to the cell wafer with UV-curable paste. A picture of a cell after dicing is shown on Fig. 5b. The 1.3 mm propagation distance needed to screen the other diffraction orders is obtained using a thickness of 1.3 mm for the gratings wafer.

### CPT measurement setup

**Figure 6:**
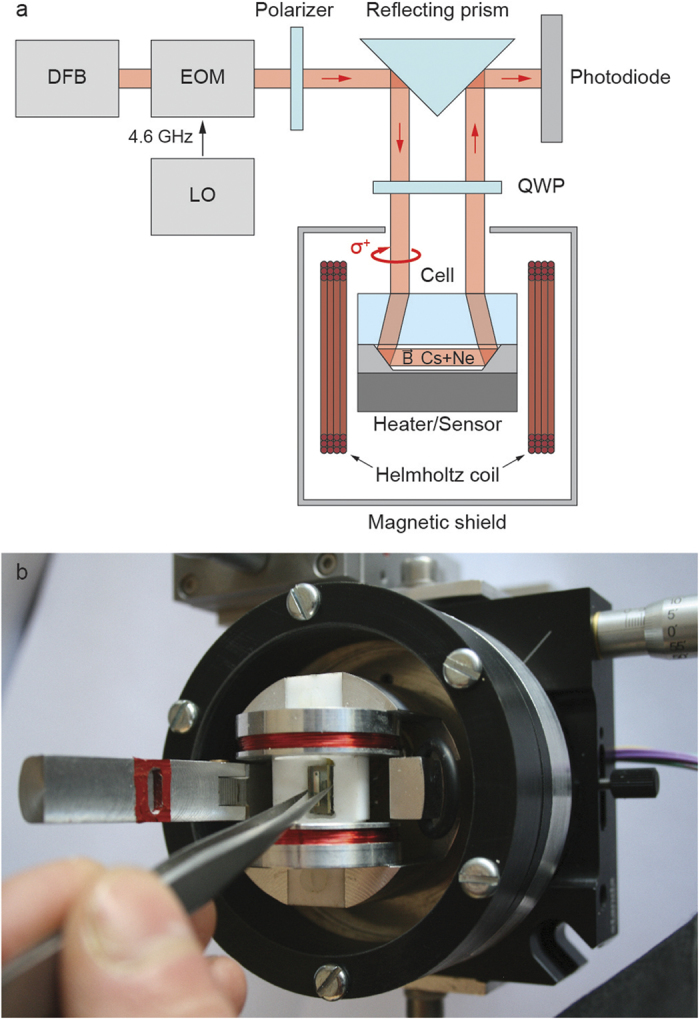
(**a**) CPT measurement setup and (**b**) dedicated physics package (magnetic shield removed).

Vapor cells were characterized through CPT spectroscopy. The measurement setup includes a distributed feedback (DFB) laser diode emitting a beam whose wavelength is tuned at 894.6 nm (Cs D_1_ line). A pigtailed Mach-Zehnder intensity electro-optic modulator (EOM) driven at 4.596 GHz by a low noise local oscillator is used to generate two first-order optical sidebands separated by 9.192 GHz, as required for CPT interaction. The optical carrier suppression at the output of the electro-optic modulator is actively stabilized by a microwave synchronous detector^41^. The light at the EOM output is then reflected by a prism coated with an aluminum layer and directed toward the cell under test. The prism can be translated to adjust the spacing between the input and the output beams according to the length of the cell under test. Before the cell, the linear polarization of the light is converted into circular polarization thanks to a QWP. The output light from the cell is reflected by the prism toward a photodiode (Fig. 6a).

The cell is inserted into a custom bench-scale physics package (Fig. 6b). The cell is heated at 75 °C, which increases the alkali atoms density and the CPT signal. This temperature is stabilized within a range of 100 μK. A small homogenous longitudinal magnetic field of 20 μT flux density is applied using a Helmholtz coil to lift the degeneracy of the ground-state Zeeman manifold. This assembly is surrounded by a single-layer mu-metal shield to protect the atoms from environmental electromagnetic perturbations. A 1 cm diameter hole in the shield provides optical access to the cell.

The local oscillator signal frequency is swept in order to detect the CPT resonance.

## Results and Discussion

### Basic theory

The short-term frequency stability of the atomic clock can be predicted from the CPT signal shape. Its Allan deviation is given by^42^:


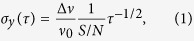


where Δ*ν* is the full-width at half-maximum (FWHM) of the resonance, *ν*_0_ is the clock frequency (about 9.192 GHz for Cs atom), *S/N* is the signal-to-noise ratio of the detected resonance in a 1 Hz bandwidth and *τ* is the integration time. The transmission contrast *C* of the CPT resonance is defined as the ratio between the signal height and the signal background. Considering only photon shot noise, the signal-to-noise ratio can be expressed as^43^:


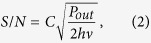


where *P*_*out*_ is the optical power reaching the photodiode and *hν* is the energy of a single photon.

As a result, the short-term frequency stability is improved by maximizing the contrast-to-linewidth ratio:


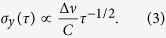


The width of the resonance depends on the time during which cesium atoms remain on the coherent dark state before relaxation. In an optically thin medium, this width is given by^44^:


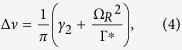


where Ω_*R*_ is the Rabi frequency, Γ^*^ is the excited state relaxation rate and *γ*_2_ is the relaxation rate of the CPT coherence.

The hyperfine coherence relaxation rate *γ*_2_ is mainly limited by three mechanisms: the collisions of Cs atoms with buffer gas atoms, the spin exchange occurring when Cs atoms collide with each other and the collisions of Cs atoms with the cell walls. This relaxation rate is expressed as^42^:


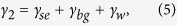


where *γ*_*se*_, *γ*_*bg*_ and *γ*_*w*_ are the relaxation rates due to spin-exchange, buffer gas collisions and cell wall collisions, respectively.

Only the latter phenomenon depends on the cell geometry. For a cylindrical cell, its rate of relaxation can be estimated by^42^:


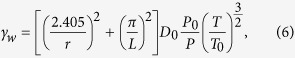


where *r* and *L* are the cell radius and length respectively, *D*_0_ (0.153 cm^2^ s^−1^) is the diffusion coefficient of Cs atoms in the Ne buffer gas^45^, *P*_0_ (101 325 Pa) and *T*_0_ (273 K) are the reference pressure and temperature, and *P* and *T* are the actual buffer gas pressure and the cell temperature.

**Figure 7:**
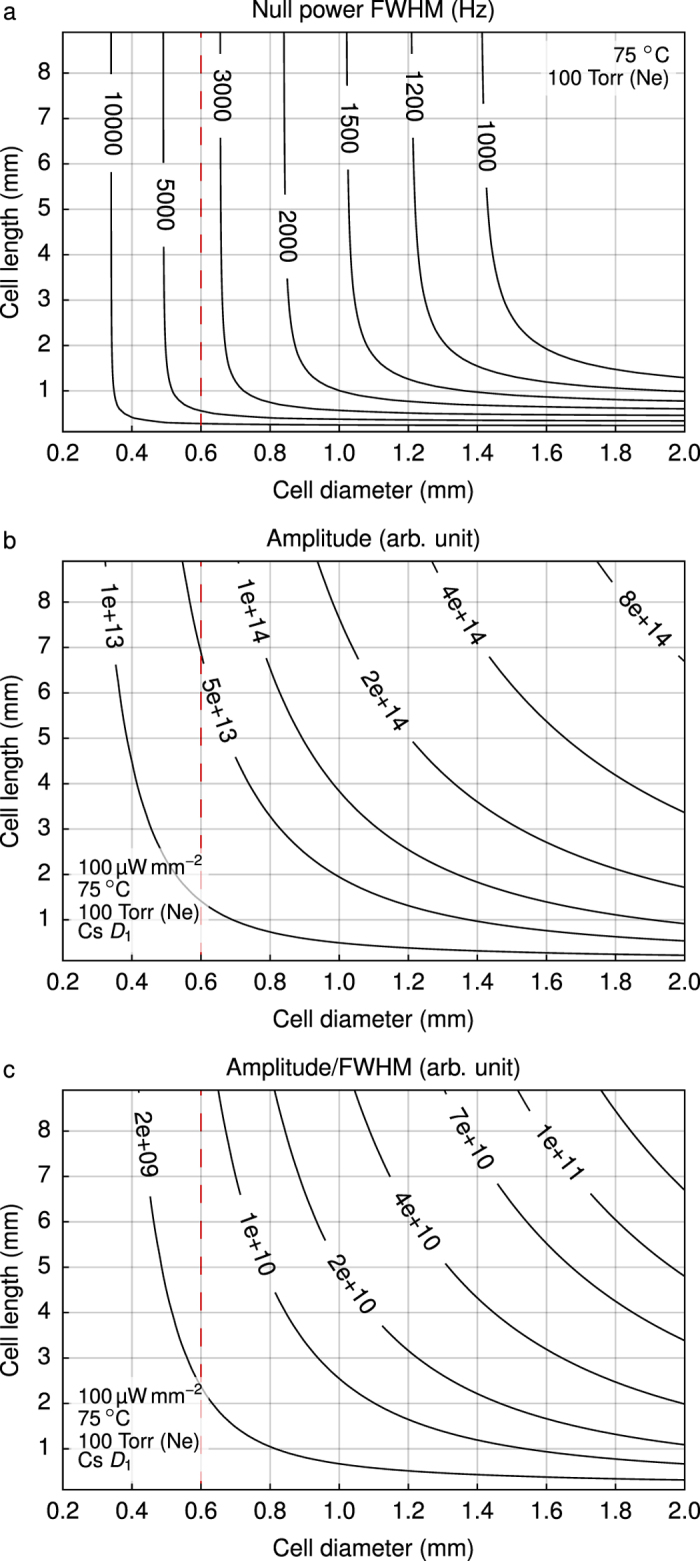
Computed (**a**) CPT linewidth at null power, (**b**) amplitude and (**c**) resulting amplitude-to-linewidth ratio of CPT signals for different cell dimensions. The cell temperature is 75 °C and the buffer gas (neon) pressure is 100 Torr.

Figure 7a reports the CPT linewidth at null laser intensity according to the cell diameter and length for a cell temperature of 75 °C and a Ne pressure of 100 Torr. It is assumed that the laser beam diameter equals the cell diameter. For a cell diameter between 0.2 and 2 mm, increasing the cell length over 2 mm does not reduce the linewidth significantly. For a 600 μm cell diameter, a 3.5 kHz linewidth is expected. This linewidth is 2.5 times higher than for a cell with a 1.5 mm diameter and 1.5 mm length, which are the typical dimensions of microfabricated cells.

The amplitude of the CPT signal is proportional to the number of atoms interacting with the field and therefore to the atomic density of cesium *n*_*Cs*_ and to the volume *V* of the cell. This amplitude can be expressed as^44^:


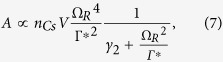


Figure 7b reports the CPT signal amplitude according to the dimensions of the cell at a laser intensity of 100 μW mm^−2^ (same temperature and pressure). The CPT signal is increased as the volume is increased. The evolution of the amplitude-to-linewidth ratio according to the cell dimensions is shown in Fig. 7c. It appears that a cell with a 600 μm diameter and a 9 mm length could provide about the same short-term frequency stability as a cell featuring a 1.5 mm diameter and a 1.5 mm length.

### Measurements

**Figure 8:**
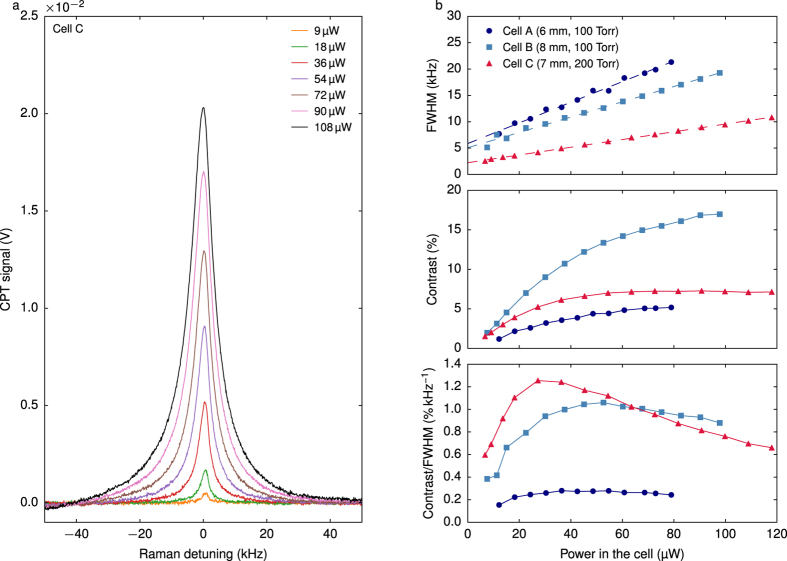
(**a**) CPT signals in cell C for different powers. The cell temperature is 75 °C and the magnetic field magnitude is 20 μT. The background is removed for clarity (**b**) linewidth, contrast and contrast-to-linewidth ratio in different cells.

Cells with different lengths were fabricated. The beam diameter was 600 μm which is defined by the gratings and apertures size. Cells A and B are filled with a Ne pressure of 100 Torr and feature lengths of 6 and 8 mm, respectively. Cell C is 7 mm long and is filled with 200 Torr. Figure 8a reports the CPT signals in cell C for different laser powers. For clarity, the background, measured to increase linearly with laser power at a rate of 0.025 V μW^−1^, is removed. CPT resonances are correctly approximated by a Lorentzian fit function from which the linewidth, the signal amplitude and the contrast are extracted. Figure 8b reports the linewidth, contrast and contrast-to-linewidth ratio obtained in the different cells at different powers. Cells A, B and C exhibit null power linewidths of 5.87 kHz, 5.07 kHz and 2.23 kHz respectively. As expected, close values are obtained for cells A and B, showing that a longer cell hardly improves the linewidth. In agreement with Equation (4), the higher buffer gas pressure in cell C reduces the null power linewidth and the laser power broadening. The null power linewidth in cell C is close to the expected value (2 kHz).

For all cells, the CPT resonance contrast increases with the laser power until saturation occurs at high laser intensities. Cell B exhibits a higher contrast than cell A, which shows that the contrast is improved as the length is increased. In particular, cell B reaches contrasts up to 17%, which is remarkable for a microfabricated cell.

In all cells, the contrast-to-linewidth ratio is maximized for a laser power lower than 60 μW. By comparing cell A and B, a longer cell tends to provide a higher contrast-to-linewidth ratio. In cell C, this figure of merit reaches 1.25% kHz^−1^ at the optimum laser power (about 30 μW) with a contrast of 5.2% and a FWHM of 4.17 kHz. Despite the stringent beam diameter reduction, this figure of merit shows a two-fold improvement compared to the reported values for microfabricated cells of this scale^10^,^19^,^40^. In this case, the estimated shot-noise limited clock short-term frequency stability is 2.9 × 10^−12^ at 1 s integration time.

### Conclusions

An alkali vapor cell based on diffraction gratings aiming at improving the integration of chip-scale atomic clocks was demonstrated. This architecture is particularly compliant with wafer-level assembly. The VCSEL and the photodiode can be integrated side by side on a single electronic plate. Because the beam diameter is reduced, a smaller distance is required to collimate the beam and the beam shaping components can be more easily batch fabricated and integrated. In addition, keeping a small cell diameter provides benefits such as the ability to realize a flat physics package, which would fit more conveniently on electronic device’s boards. Such features can be the keys to cost and size reduction of atomic clocks without compromising performances. Indeed, this architecture allows to increase the length of the cell which effectively compensate for a reduced beam diameter. The achieved figure of merit can even be higher than for conventional microfabricated cells in which the beam diameter is kept equal to the cell length. This result was not straightforward. Indeed, an elongated cell does not allow the reduction of the CPT resonance linewidth because of the dominating sidewalls collision rate. However, thanks to the significantly improved contrast, the performances of these cells are comparable despite a one-half volume reduction. In the future, thermal and magnetic aspects will be addressed to achieve a complete miniature physics package.

The architecture presented here could also be of interest in other atomic devices including magnetometers^46^,^47^, gyroscopes and, more widely, in any sensor requiring to probe a volume optically without changing the polarization state (circular or crossed linear). In this respect, the same cells can be employed with various pumping polarization schemes such as push-pull^41^ or lin_┴_lin^48^ leading to higher resonance contrasts. Laser cooling experiments could also benefit from this architecture^49^.

## Additional Information

**How to cite this article**: Chutani, R. *et al.* Laser light routing in an elongated micromachined vapor cell with diffraction gratings for atomic clock applications. *Sci. Rep.* **5**, 14001; doi: 10.1038/srep14001 (2015).
